# Concussion vs. resignation by submission: Technical–tactical behavior analysis considering injury in mixed martial arts

**DOI:** 10.3389/fneur.2022.941829

**Published:** 2022-08-18

**Authors:** Bianca Miarka, Dany A. Sobarzo Soto, Esteban A. Aedo-Muñoz, Clóvis A. Maurício, Vanessa T. Müller, Nicola L. Bragazzi, Ciro José Brito

**Affiliations:** ^1^Laboratory of Psychophysiology and Performance in Combat and Sports, Physical Education Postgraduate Program, Federal University of Rio de Janeiro, Rio de Janeiro, Brazil; ^2^Escuela de Kinesiología, Facultad de Salud, Universidad Santo Tomás, Puerto Montt, Chile; ^3^Department of Physical Education, Sports and Recreation, Universidad Metropolitana de Ciencias de la Educación, Santiago, Chile; ^4^Department of Mathematics and Statistics, Laboratory for Industrial and Applied Mathematics, York University, Toronto, ON, Canada; ^5^Physical Education Postgraduate Program, Juiz de Fora Federal University, Juiz de Fora, Brazil

**Keywords:** neurology, trauma, time and motion studies, risk factors, injury, rehabilitation

## Abstract

This research study verified the technical–tactical actions during the mixed martial arts (MMA) fights to generate serious enough injury to stop the MMA round, determining technical-tactical potential risk factors for injury in official MMA combats, according to Concussion or Resignation per Submission. A total of 990 rounds with concussions and 627 resignations per submission were considered with severe injury (i.e., a fight ended by a doctor or referee). All injuries were diagnosed and managed by attending ringside physicians during the MMA fights and rounds and had a continuous technical–tactical behavior analysis, *p* ≤ 0.05. The leading cause of concussion was due to head trauma (~90%), with higher dependence on head strikes scored actions. Comparisons between Concussion and Resignation per Submission combats demonstrated differences between distance head strikes actions [13(6,25) vs. 9(4,18) frequencies], clinch head strikes actions [1(0;4) *vs*. 1(0;3) frequencies], ground head strikes actions [1(0;8) vs. 2(0;10) frequencies] and takedowns actions [0(0;1) *vs*. 1(0;2) frequencies]. This information may provide significant evidence regarding the doctor stoppage in concussion combats and when it could be called by officials supervising MMA.

## Introduction

Technical–tactical behavior analysis has been applied to study actions during mixed martial arts (MMA) tournaments and produced accurate recordings of movement patterns during this combat modality (1, 2). MMA fights are typically decided using strategic methods with an intentional goal to cause a discontinuance of the combat either by submission (verbal or physical signal of wish to discontinue the combat), knockout (KO), or technical knockout (TKO) due to judge decision after an allotted amount of time has elapsed (3). Fights may also end because of athlete retirement, forfeit, disqualification, or TKO by doctor stoppage (4, 5). The specific actions during the rounds are considered acyclic with open tasks involving aggressive strikes (punches, kicks, knees, and elbow attacks), as well as grappling actions (tackles, twists, and projections using legs) and submissions on the ground, such as chokes and joint locks (6) that can produce acute and chronic severe injuries (7, 8). Studies regarding the mechanism and seriousness of injuries (9) predominantly connected with technical-tactical actions are essential to understanding the interaction of these variables with a severe injury during MMA combats (7, 10).

There has been increased interest in concussion incidence (10–13) risk factors (14) and types of injuries in MMA (15, 16). Preceding results have indicated that the incidence of head traumas ranges between 58 and 78% of all injuries (17), highlighting maxillofacial traumas (18, 19). Studies also have reported all-cause injury incidence rates ranging from 23.6 in 100 fight participations to 28.6 in 100 fight participations in MMA (20). These values are equivalent to reports for taekwondo (4, 21, 22), karate (23, 24), kickboxing (25, 26) and boxing (27, 28). Bernick et al. (29) indicated a mean of 0.061 concussions (0.047 for boxers and 0.085 for MMA) per minute of combat time.

Concussions are present in different sports, as the pooled incidence of concussions per 1,000 younger athlete exposures (AEs) across 12 sports (football, rugby, hockey, lacrosse, soccer, basketball, baseball, softball, wrestling, field hockey, track, taekwondo, volleyball, and cheerleading) in 13 studies using a random-effects model was 0.23 (95% CI 0.19 to 0.28) (30). The three sports with the highest incidence rates were rugby (4.18 AEs), hockey (1.2 AEs), and American football (0.53 AEs) (30). Lowest incidence rates occurred in volleyball (0.03 AEs), baseball (0.06 AEs) and cheerleading (0.07 AEs). Long-term clinical cognitive consequences were shown, as 1,513 retired professional football players had ≥1 and 597 ≥ = 3 concussions with a prevalence of mild cognitive impairment (31). Moreover, in comparing former university athletes with 24.00 ± 4.55 concussions vs. the control group with 0 concussions, former athletes showed reduced semantic verbal fluency, reduced recognition, and delayed recall (32). In addition, after a concussion ending-fight, despite the indications in the rules of striking combat to prevent risks associated with concussion, ~40% of amateur fighters reported returning to competition or training on the same day a head trauma was sustained. This percentage is double that found in boxing, as ~20% of boxers endorsed hiding symptoms of head trauma from coaches and medical providers (27). However, grappling athletes had a high concussion prevalence, Brazilian jiu-jitsu demonstrated 25.2% (33) and wrestling had a 19.5% of concussion prevalence (34) during training and tournaments. In terms of injury severity patterns, a consecutive case series using professional data from post-fight medical checkups recommended that knockouts (KOs) and TKOs in MMA seem to be connected with a long time of post-combat medical suspensions and brain trauma reports (7, 35).

The fighters in MMA are constantly projecting, kicking, or striking at each other with extreme power to debilitate their opponent and claim victory. Considering such a high participation rate in MMA (4), athlete safety is the highest priority. Moreover, the moments of exposure during the bout in which fighters become more susceptible to trauma are unknown during combats ended by concussion or resignation by submission. Previous studies have observed injury incidence in MMA and found lacerations and concussion head injuries to be the most common MMA injuries (4, 9, 35). Although the concussion occurs in striking and grappling actions, the possibility of ending the round by resignation instead of targeting the head is essential to reduce the possibility of chronological effects of concussions in athletes. In addition, performance analysis of MMA combats can demonstrate technical variability in what makes the MMA event commercial.

At most competitions, the medical staff stands on hand to manage care for athletes who suffer injury. This effort at making violent, inherently unsafe combat as safe as possible has stopped abundant competitors from suffering trauma (4). These devastating injuries have been inflicted because an injured athlete can continue fighting (4, 35). A TKO by doctor stoppage occurs when a fight is stopped through a subjective analysis because of a competitor's inability to logically or safely defend himself/herself (36). Preceding rates of KOs and TKOs have drawn the attention of the scientific community (37) given the probably debilitating acute and chronic effects associated with concussions and repetitive traumatic brain trauma (9, 26). To date, no one knows a real enough number of strikes for a doctor to stop TKO in professional fights. This analysis could reduce acute and chronic injury caused by excessive attacks, particularly in the head (12, 38).

Shin et al. (39) scanned 155 boxing and mixed martial arts fighters using diffusion tensor imaging (DTI) and demonstrated that the number of KOs in MMA athletes is prognostic of microstructural injury in the brain. Chronic traumatic encephalopathy was recently associated with MMA after a middle-aged male case exhibited memory impairment, mood, and behavioral changes after years of competition (7, 27).

In contrast, Stellpflug et al. (40) described an analysis of 5,834 fights in 538 Ultimate Fighting Championship™ (UFC™) events, finding 1,186 fight-ending grappling submissions (chokes and non-chokes) and 904 fight-ending choke holds. The 904 fight-ending choke holds comprise 15.5% of the total fight outcomes and 76.2% of the total grappling submissions. Almost half (444–49.1%) of the chokes were rear-naked chokes, and most fight-ending chokes culminated in voluntary submission. Preceding studies have also suggested a relation between chronic traumatic encephalopathy and repeated traumatic brain injuries in striking combats, such as boxing, taekwondo, and kickboxing (9, 11). There is substantial debate over appropriate preventive approaches to decrease the burden of traumatic brain injuries in MMA fighters (7, 9, 28). Thus, this study aimed to verify actions associated with concussion and resignation by submission (locks and chokes) during MMA fights, which could generate serious enough injury to stop the fight.

## Methods

### Study design

This retrospective epidemiological study adhered to the guidelines of the Strengthening the Reporting of Observational Studies in Epidemiology Statement (41). We created a protocol (Table 1) based on previous studies that assessed injury factors (8, 20, 42) and behavior technical-tactical patterns (1, 43, 44), which were incorporated into the assessment and validation analysis (45, 46). Next, a stratified selection and comparison of MMA rounds with and without injury were done in the MMA championships (6, 24, 47). The study conforms to the World Medical Association Code of Ethics (approved by the local ethics advisory board).

**Table 1:** Criteria of technical-tactical analysis from MMA performances and intraclass coefficient of correlations.

**Behavior analysis**	**Conditions of analysis**	**ICC (r)**	**Reliability**
Strikes actions	Any action to carry out an attack with striking movements (punches, kicks, elbows and knees) at the adversary.	1.00	Excellent
Head strikes actions	Any action to carry out an attack to the adversary's head with striking movements (punches, kicks, elbows and knees).	1.00	Excellent
Body strikes actions	Any action to carry out an attack to the adversary's trunk or arms with striking movements (punches, kicks, elbows and knees).	0.98	Excellent
Leg strikes actions	Any action to carry out an attack to the adversary's leg with striking movements (punches, kicks, elbows and knees).	0.97	Excellent
Takedowns actions	An action that involves off-balancing an adversary trying to bring him to the ground, typically with the attacker landing on top.	0.92	Excellent
Submission actions	It occurs when one of the athletes tries to dominate the adversary on the ground, keeping his opponent with one or both shoulders on the ground.	0.94	Excellent
Locks actions	Lock action involving manipulation of an adversary's joints in such a way that the joints reach their maximal range of motion.	0.90	Excellent
Chokes actions	Any action to do a mechanical obstruction of the flow of air into the lungs of the adversary.	0.83	Good

### Participants

The present study considered 1,617 MMA rounds stratified from events between 2014 (after anti-doping policies increased, followed by the introduction of the U.S. Anti-doping Agency as the official anti-doping agency of the UFC™ in 2015) and 2019 (before the Pandemic COVID-19) of 635 athletes from UFC™ championships. The present study stratified all rounds from the total amount that resulted in severe injury considering a fight stoppage performed by a doctor or referee. A total of 990 rounds with concussions and 627 resignations by submission combats were considered with severe injury. The inclusion criteria were to have a round ending with resignation by submission or concussion by KO, TKO, or doctor stoppage during the MMA fight. The videos were obtained and selected at https://www.ufc.com.br/watch/library. All records had sufficient quality with high definition and were taken from a landscape view of the complete UFC™ octagon. Exclusion criteria concerned matches in which the end of the fight was a draw or no result, or finished due to unforeseen circumstances such as illegal actions. The study was previously approved by the local ethics (2014/61) and research committee and completed within the guidelines set forth in the Declaration of Helsinki.

### Procedures and measurements

All injury was diagnosed and managed by attending ringside physicians during the MMA fights, as the injury was recorded at the discretion of attending ringside doctors (15). Injury described in the official event records were coded according to the Orchard Sports Injury Classification System (OSICS, version 10.8), following previous reports (5, 48). The fights were analyzed using a previously developed protocol (9, 12) (Table 1) by five analysts who determined the frequency of striking and grappling actions (6, 49–51). There is typically a mean (min-max) of 2 (1–5) rounds with 264 s (6–315 s) in an MMA fight (52–54).

For performance analysis, two experts in Mixed Martial Arts analyzed 10 videos randomly selected from an amount of 635 MMA combats that were analyzed and reanalyzed 24 h later. All evaluators had ≥20 years of experience and competed at the national level. The inter-observer reliability correlation between measurements obtained for each model was verified by the interclass correlation (ICC) (2). A preceding study (55) indicated the following criteria for ICC inter-rater agreement measures: Less than.40 as poor; between.40 and.59 as fair; between.60 and.74 as good; and between.75 and 1.00 as excellent. The inter-observer reliability correlation between measurements obtained an agreement classified as “excellent” for all variables, and is shown in Table 1. Video analyzes were performed after reliability results, following criteria described in Table 1.

### Statistical analysis

The Kolmogorov–Smirnov test (K–S) was used to determine the normal distribution of the data. Descriptive data of frequency of dependent variables are presented as median, first quartile (1Q), and third quartile (3Q). The Mann–Whitney test was applied for the non-parametric data to compare the effects of Concussion vs. Resignation by Submission combats. Afterward, the effect size for non-parametric analysis was calculated, defined as ES = Z/√N, where ES represented the effect size, Z was derived from the conversion of the Wilcoxon test, and N was the total number of observations. The analysis considered ES-values as small (ES < 0.10), medium (ES < 0.30), or large effect size (ES < 0.50). The significance level of *p* ≤ 0.05 was used. All analyses were conducted using the SPSS 20.0 program for Windows.

## Results

No effects were observed for severe trauma (excluding concussion and the resignation submission) between Concussion and Resignation by Submission combats (*p* = 0.155). In concussion combats, 0.2% demonstrated ankle injury, 0.4% knee injury, 0.8% leg injury, 0.2% rib injury, and 0.4% arm injury, while resignation by submission combats showed 0.3% of calf slicer, 0.3% of face crank, 0.3% of straight armbar injury, 0.3% standing rear-naked choke injury, 5.7% undefined submissions injury, 0.6% triangle armbar injury, and 0.3% of von flue choke injury.

From the total concussion vs. resignation by submission combats, 36.6 vs. 35.2% ended during the 1st round, 38.6 vs. 36% ended during the 2nd round, and 24.8 vs. 28.7% ended during the last round. No effects were observed in the ending round comparison (*p* = 0.214) between concussion and resignation by submission combats. Table 2 shows striking and grappling actions: round comparisons of Concussion and Resignation by Submission groups by each round.

**Table 2:** Striking and grappling actions compared by concussion and resignation per submission groups by round.

**Action/round**	**Concussion**			**Resignation per submission**			**Inferences**		
	**Med**	**Q1**	**Q3**	**Med**	**Q1**	**Q3**	***p*-value**	***U*-value**	**ES**
Distance head strikes scored	4	1	7	2	1	6	≤0.001	257906	0.15
Distance head strikes attempted	13	6	25	9	4	18	≤0.001	255828.5	0.15
Distance body strikes scored	1	0	2	0	0	2	≤0.001	282131.5	0.08
Distance body strikes attempted	1	0	3	1	0	2	≤0.001	280,276	0.09
Distance leg strikes scored	1	0	2	0	0	1	≤0.001	276,625	0.10
Distance leg strikes attempted	1	0	3	1	0	2	≤0.001	280,489	0.09
Clinch head strikes scored	0	0	2	0	0	2	0.005	286787.5	0.06
Clinch head strikes attempted	1	0	4	1	0	3	0.013	289,011	0.04
Clinch body strikes scored	0	0	2	0	0	2	0.76	307889.5	0.01
Clinch body strikes attempted	0	0	2	0	0	2	0.93	309708.5	0.02
Clinch leg strikes scored	0	0	0	0	0	0	0.12	301004.5	0.04
Clinch leg strikes attempted	0	0	0	0	0	0	0.13	301195.5	0.04
Ground head strikes scored	1	0	6	1	0	8	0.045	327,865	0.05
Ground head strikes attempted	1	0	8	2	0	10	0.066	326,531	0.05
Ground body strikes scored	0	0	0	0	0	1	≤0.001	343,142	0.12
Ground body strikes attempted	0	0	0	0	0	1	≤0.001	342,811	0.12
Ground leg strikes scored	0	0	0	0	0	0	0.68	311590.5	0.01
Ground leg strikes attempted	0	0	0	0	0	0	0.76	311,308	0.01
Takedowns scored	0	0	0	0	0	1	≤0.001	363616,5	0.18
Takedowns attempted	0	0	1	1	0	2	≤0.001	3557847	0.14

Table 3 shows submission actions: round comparisons of Concussion and Resignation by Submission combats.

**Table 3:** Submission actions compared by concussion and resignation per submission groups.

**Variables/Group**	**Percentage of frequencies/ round**					**Inferences**			
	**0**	**1**	**2**	**3**	**4**	**5**	***p*-values**	***U*-values**	**ES**
**Resignation per submission**
Submission attempted	61.40%	29%	7.3%	1.8%	0.2%	0.2%	≤0.001	409,500	0.40
Chokes attempted	67.8%	26.8%	4.6%	0.8%	0%	0%	≤0.001	397,096	0.39
Locks attempted	89.6%	8.8%	1.1%	0.5%	0%	0%	≤0.001	333,156	0.16
**Concussion**
Submission attempted	93.2%	5%	1.2%	0.1%	0.1%	0%	≤0.001	409,500	0.40
Chokes attempted	95.7%	3.8%	0.4%	0.1%	0%	0%	≤0.001	397,096	0.39
Locks attempted	97.0%	2.6%	0.3%	0.1%	0%	0%	≤0.001	333,156	0.16

Regarding the ending target, significant differences were observed between concussion and resignation by submission combats (U = 10335.00, *p* ≤ 0.001), concussion combats demonstrated that 90.7% of all combats had the head as the final target, 2.6% undefined target, 2.6% body, and 0.4% leg, while the resignation by submission group had 99% of undefined target, 0.3% body, and 0.6% target on the head (*p* ≤ 0.001). Table 4 shows the ending techniques associated with concussion or resignation by submission combats.

**Table 4:** Ending technique in frequency, percent and cumulative percent, according to concussion or resignation per submission groups.

**Actions**	**Frequency**	**Percent**	**Cumulative percent**
**Concussion**
Undefined	26	2.6	2.6
Elbow	17	1.7	4.3
Elbows	67	6.8	9.4
Flying Knee	10	1	10.4
Kick	92	9.3	19.7
Kicks	4	0.4	20.1
Knee	56	5.7	25.8
Knees	12	1.2	27
Punch	447	45.2	72.1
Punches	270	27.3	99.4
Slam	2	0.2	99.6
Spinning back kick	4	0.4	100
**Resignation per submission**
Anaconda choke	8	1.3	1.3
Arm triangle	58	9.3	10.5
Armbar	66	10.5	21.1
D'Arce choke	22	3.5	24.6
Guillotine choke	143	22.8	47.4
Heel hook	4	0.6	48
Kimura	14	2.2	50.2
Kneebar	8	1.3	51.5
Neck crank	2	0.3	51.8
North-South choke	2	0.3	52.2
Omoplata	2	0.3	52.5
Other-choke	2	0.3	52.8
Other-lock	6	1	53.7
Rear naked choke	226	36	89.8
Strikes	8	1.3	91.1
Triangle choke	56	8.9	100

## Discussion

The present study's primary purpose was to describe the injury aspects and action demands of MMA rounds with particular reference to rounds stopped by concussion or submission due to injuries determined by the ringside doctor. The main descriptive results in concussion combats showed that ~90% of fight stoppages were caused by head trauma, with striking head actions being the leading cause of all injuries, while the submissions group had 0.6 fights finished from head trauma. The descriptive analysis demonstrated a prevalence of head and neck injury, with the potential for severe injuries in combats finalized by concussion, particularly to the brain, which is persistent in MMA (56). Significant effects were observed between groups in takedowns, and distance and ground striking actions.

The present trauma results of MMA concussions indicated the need for concussion rules changes and, if it occurs, monitoring the recovery with biomarkers. Concussion rounds had ~60% more striking actions than resignation by submission. There could be a maximum count of distance or sequential head strikes attacks by round in MMA rules, this brings more dynamism to the MMA fight because the athletes would have to make attacks in different directions (e.g., legs or body), more defenses and improve their reaction time. Moreover, after concussion occurrence, athletes have to be neurologically monitored (57). Bishop and Neary (57) assessed prefrontal cortex oxygenation after concussion using near-infrared spectroscopy, which showed oxygenation changes in the brain. Previous research also demonstrated that MMA fighters exhibited reduced concentration, memory, and processing speed relative to the control group in neuropsychological testing coupled with a decrease of thinning in the left middle and superior frontal gyrus and reduced cortical thinning in the left posterior cingulate gyrus and right occipital cortex (7, 38, 58). Post-fighting scores were expressively worse for fighters with head trauma during the fight (17). A past study found anomalies in MMA fighters having different brain structures, but it seems that the thalamus and caudate are the most affected (17). The reduced performance in verbal memory, processing speed, and psychomotor speed is regularly established in investigations with fighters (17, 47). Head trauma could be a risk factor for the development of neurodegenerative conditions (11, 17, 26, 39, 56), and it may be one of the probable causes of chronic traumatic encephalopathy (16). The present results can be used to reduce the impact caused by more harmful techniques.

Concussions and blunt force trauma are a significant concern in contact and combat sports (36, 47, 59). One study detected a higher incidence of concussions involving loss of consciousness in MMA athletes and boxing athletes (~4 and ~7%) (35). Studies have shown that musculoskeletal injuries such as sprains, dislocations, and fractures in other combat sports, such as taekwondo (5), karate (24), wrestling (4), and judo (42) with fewer injury coming from lacerations, abrasions, cuts, or epistasis which induce blood loss than MMA data which demonstrated ~80% of injury with lacerations (8).

Elite athletes demonstrate 2–5 times more traumatic injuries than non-elite fighters (24). This result is probably due to helmets and the prohibition of specific techniques such as elbow strikes (22). Previous research also shows that striking sports have a typical distribution of injury by anatomic region (4, 15) and is similar to that demonstrated in our study. The head/neck was the most frequently injured anatomical region in MMA (~65%), boxing (~85%), karate (~75%), and kickboxing (~55%), whereas the lower and upper limbs were the most common anatomical regions in taekwondo (~50%) and judo (~50%) (15). Head trauma in MMA has also been associated with temporomandibular disorders because of the intensity and duration of training needed for professional competitions (18, 19).

Upper eyelid and eyebrow lacerations of fighters are recurrent and troublesome during MMA fights given the effect of hemorrhage from facial injury on the fighter's vision and in turn their ability to continue fighting (48). Findings have demonstrated one mutual action among all injury cases: head strike scoring actions. This data reveals that many basic techniques could be essential to winning, but under combat conditions and are related to severe injury (60). A past study indicated that injuries in doctor stoppage situations occurred in specific attacks that emerged when the fighter performed groundwork combat with an increase in the submission, lock, and choke actions (8). Thus, strength and conditioning coaches should be aware of the increase in the frequency of critical actions from the technical-tactical round differences, especially of striking defenses, and takedown training could focus on high-speed defenses and attacks while in a fatigued state, simulating the metabolic demands and tactical necessities of the final round (1, 2). Previous research indicates that the most common conclusion to MMA combat is a technical knockout followed by a submission (14).

Regarding limitations of the present study, it only included data from MMA contests sanctioned by UFC™ fights, limiting the generalizability of the reported data. This study did not adopt a strict operational injury definition for pragmatic reasons, following previously published research (9, 48). Moreover, the injury recording was at the discretion of the attending ringside physicians. Therefore, it is possible that several traumas that occurred were not recorded (48). This fact may result in underestimating the actual risk of severe injury, while potentially overestimating the relative proportion of more severe injuries, such as fractures. Indeed, knowledge of the present results is essential to establish technical and tactical strategies, as defeated fighters have 3 × more risk of injury than winners, and athletes in fights ending by KO or TKO have 2 × the risk of injury as fighters in fights ending in submission (20, 48). Moreover, the present data agree with the previous findings regarding the incidence of injury sustained in MMA and boxing (4, 28, 36). As MMA rules have to be constantly concerned with greater emphasis on safety (3, 4), supplementary priority could be given to maneuvers that do not involve high concussion risk and blow to the head. Furthermore, studies on the impact attenuation of protective headgear in martial arts did not support this suggestion (61). Indeed, the headgear may increase aggressive fighting (15, 62–65), and it could increase judge tolerance before stopping a fight.

This study focused on verifying what kind of technical and tactical actions occurred during the rounds to generate serious enough injury to stop the fight. With this data, it is possible to summarize how and when fighters are predisposed to severe injury in MMA, as well as to develop suggestions for rules which may allow a technical knockout to be declared based on an objective performance analysis measurement, such as a specific number of unrequited attacks per round time.

## Conclusion

The information gained from this study enhances the mechanisms of injury during an MMA fight and the situational factors involved. Concussions associated with blunt force trauma to the head were the main worries in MMA fighters, and the present data demonstrated that more than 90% of serious injuries occurred to the head, with striking head actions, such as sequential kicks, elbows, isolated knee, and punches, being the main factors. The rates of strikes on the head per round are higher than those reported in other combats; this outcome is associated with the risk of brain injuries, and severe injury provoking doctor stoppage, as the athlete does not show cognitive and physical function to continue the combat. These findings suggest a rule change to stimulate grappling actions instead of targeting the opponent's head. Future advances in MMA injury prevention will only be achieved if study efforts are concentrated on understanding the implementation context (i.e., technical–tactical analysis) for injury prevention and continuing to build the database for the efficacy and effectiveness of training interventions.

## Data availability statement

The raw data supporting the conclusions of this article will be made available by the authors, without undue reservation.

## Author contributions

All authors listed have made a substantial, direct, and intellectual contribution to the work and approved it for publication.

## Funding

This study was financed in part by the Fundação de Amparo à Pesquisa do Rio de Janeiro (FAPERJ) [Grant# E# 26/202.810/2019(247397)].

## Conflict of interest

The authors declare that the research was conducted in the absence of any commercial or financial relationships that could be construed as a potential conflict of interest.

## Publisher's note

All claims expressed in this article are solely those of the authors and do not necessarily represent those of their affiliated organizations, or those of the publisher, the editors and the reviewers. Any product that may be evaluated in this article, or claim that may be made by its manufacturer, is not guaranteed or endorsed by the publisher.
